# Building research capacity for sickle cell disease in Africa: Lessons and challenges from establishing a birth cohort in Tanzania

**DOI:** 10.3389/fped.2022.826199

**Published:** 2022-09-09

**Authors:** Siana Nkya, Belinda J. Njiro, Doreen Ngowi, David Solomon, Frida Kaywanger, Salama Nyangasa, Godfrey Ndoje, Emmanuela Marco, Mazoea Moses, Julie Makani

**Affiliations:** ^1^Department of Haematology and Blood Transfusion, Muhimbili University of Health and Allied Sciences, Dar es Salaam, Tanzania; ^2^Biological Sciences, Dar es Salaam University College of Education, Dar es Salaam, Tanzania; ^3^Tanzania Human Genetics Organization, Dar es Salaam, Tanzania

**Keywords:** Sickle Cell Disease, newborn screen (NBS), birth cohort, immunization, genetics

## Abstract

Sickle Cell Disease (SCD) is a known public health burden in sub-Saharan Africa (SSA). The manifestation of SCD starts in early childhood and if not well-managed may lead to early death (before the age of 5 years). Understanding the underlying mechanisms that influence early SCD manifestation is of great importance for early disease and intervention management which will in turn, reduce both morbidity and mortality rates in children. One approach of achieving this is by establishing SCD birth cohorts that can be followed for a period of time (3–5 years) whilst documenting necessary information related to early childhood illnesses. To date, there are few SCD birth cohorts in Africa. To address this gap, we have established a birth cohort of babies with and without SCD (with sickle cell trait and healthy babies). These babies are followed up for 3 years with their study visits synchronized to the immunization schedule. During enrollment and follow-up visits, information on demographic, clinical, and laboratory parameters are collected. To date, we have enrolled a total of 341 babies with and without SCD. Out of these, a total of 311, 186, 133, 81, 44, and 16 babies have returned for their 1st, 2nd, 3rd, 4th, 5th, and 6th visits, respectively. We have collected both demographic and clinical information for these babies at enrollment and during follow-up. We have also utilized this platform to learn on the best approaches of establishing and maintaining a research birth cohort in an African context. We have analyzed the practical issues pertaining to the integration of the birth cohort with the immunization platform which seems to be the most effective and sustainable strategy for maintaining a birth cohort in our context.

## Introduction

Sickle cell disease (SCD) is a global public health issue and calls for global initiatives which can also work in the local contexts. In 2010, the annual global estimate for children born with SCD was 300,000. This number is expected to increase to 404,200 in 2050 (1). The burden is bigger in SSA and India where there is severe morbidity and high mortality among children. Early-life mortality in Africa ranges between 50 and 90% of children born with SCD (2). In most of tropical African countries, including Nigeria, Congo and Cameroon, SCD has not yet received much attention compared to other diseases such as malaria, malnutrition, HIV/AIDS, and other neonatal illnesses (3, 4).

Early screening of SCD either at birth or through the first immunization clinic has proven to be a game-changer associated with early disease management and reduced mortality and morbidity of up to 75% (3). There are no universal screening programs for SCD in Africa, however, few countries including Ghana, Nigeria, Angola, Uganda, and Tanzania have conducted newborn screening (NBS) programs covering some parts of their countries (4–6). Comprehensive care for these newborns and educational programs for their families have been proven to improve the health of the babies. To date, there is a strong push toward the establishment of universal NBS programs for SCD, especially in low resource countries such as those in Africa (7). These NBS programs are not only important for early diagnosis and comprehensive care provision but are also utilized to generate data for the cost-effectiveness of NBS and early interventions for SCD in SSA (7–9). In Tanzania for example, it is estimated that more than 11,000 babies are born with SCD each year and without proper intervention, these children will die before reaching adulthood (10, 11). These estimates are based on the partial NBS programs conducted in the country. To date, Tanzania has conducted NBS in the capital city, Dar es Salaam through the support of donors including the Foreign, Commonwealth & Development Office, UK and the American Society of Hematology (ASH) and the National Institute**s** of Health (NIH), USA.

Following NBS initiatives, there is a great opportunity to establish birth cohorts that can be followed up with the aim of investigating and understanding childhood illnesses and the best management strategies. This is especially important for babies with SCD who are more vulnerable to the causes of under-five mortality. SCD birth cohorts can also serve as platforms for planning appropriate interventions both at childhood and adult levels. Despite their importance, the establishment of such birth cohorts faces similar challenges to the establishment of NBS in Africa mainly due to limited resources, including human, financial and logistical.

One approach to address these challenges is to learn and integrate such cohorts with successful childhood programs. National immunization programs are among the most successful healthcare programs in the world. In Tanzania for example, the immunization program is described to be among the best performing programs in Africa (12) with more than 95% of babies being vaccinated as scheduled following the “Reach Every Child” approach. Therefore, establishment of research birth cohorts can utilize such platforms in order to tap into the existing resources which will ensure increased sustainability of the research interventions and outcomes. According to the Tanzania Expanded Program of Immunization (EPI), vaccines for under-five children are provided at birth, 6 weeks, 10 weeks, 14 weeks, 9 months, and 18 months of age (Table 1) (13).

**Table 1:** Immunization schedule for under-five children in Tanzania (Adapted from the Expanded Program of Immunization (EPI), Tanzania) (13).

**Age**	**Vaccine**
At birth	BCG, OPV 0
6 weeks	OPV1, DTP-HepB-Hib1, PCV1, Rota 1
10 weeks	OPV2, DTP-HepB-Hib2, PCV2, Rota 2
14 weeks	OPV3, DTP-HepB-Hib3, PCV3
9 months	MR 1
18 months	MR 2

BCG, Bacillus Calmette-Guerin; DPT, Diphtheria, Pertussis, Tetanus vaccine; HepB, Hepatitis B vaccine; Hib, Hemophilus Influenzae type B vaccine; PCV, Pneumococcal Vaccine; Rota, Rotavirus vaccine.

OPV 1, 2, 3, OPV type 1, type 2 and type 3 Oral Poliovirus Vaccine respectively.

OPV0, zero dose Oral Polio Vaccine.

DTP-HepB-Hib1, PCV1, first dose of DTP, HepB, Hib and PCV.

DTP-HepB-Hib2, PCV2, second dose of DTP, HepB, Hib and PCV.

DTP-HepB-Hib3, PCV3, third dose of DTP, HepB, Hib and PCV.

MR1, Measles and Rubella virus vaccine first dose.

MR2, Measles and Rubella virus vaccine second dose.

From 2019, The Muhimbili University of Health and Allied Sciences (MUHAS) through the support from Fogarty International Center Emerging Global Leader Award, has established a birth cohort of babies with and without SCD. The main purpose of this cohort is to investigate the genetic determinants of fetal hemoglobin decline and how this phenomenon may influence the clinical manifestation of SCD in the first 3 years of life. The birth cohort is followed for the first 3 years of life utilizing the immunization platform through (i) synchronizing study visits with the national immunization clinics, (ii) utilizing the expertise of nurses at the immunization clinics in providing health education/counseling, and (iii) sharing logistical/infrastructural resources. In this report, we share the lessons, challenges, and experiences of establishing a birth cohort in Tanzania. We believe that most of the lessons can be applied to any African setting as well as other countries with limited resources.

## Materials and methods

### Study design and sites

**Figure 1:**
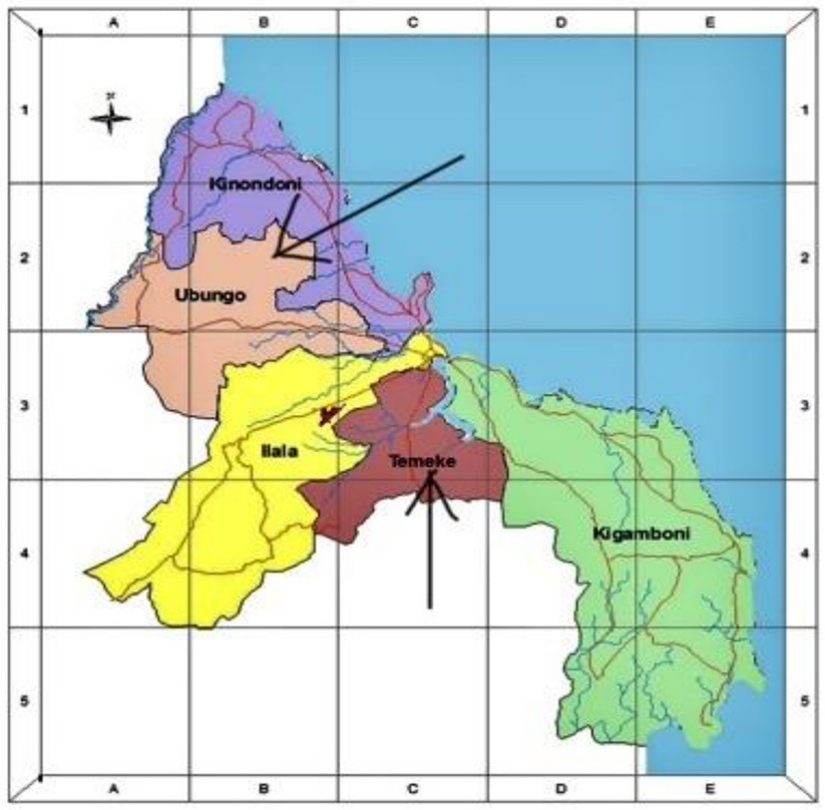
Map of Dar-es-Salaam region showing the study sites (Ubungo and Temeke Municipalities).

This is a prospective study involving a cohort of newborns born at Mbagala and Sinza hospitals from 2019 to 2021. It is a part of a larger project on the “role of fetal hemoglobin decline and its determinants on Sickle Cell Disease expression in the first three years of life.” The study will involve both babies with and without SCD. Both Mbagala and Sinza hospitals are classified as district hospitals in the Temeke and Ubungo municipalities in Dar es Salaam region (Figure 1). These health facilities are designed to offer inpatient and outpatient services to a large number of people from diverse social backgrounds. Provided services include but are not limited to women's health, labor & delivery, counseling and minor/major operating theaters which are required settings for the practicum experience. In addition, both facilities were selected due to their designation for providing immunization services for newborns residing in their catchment areas.

### Study population

This study involved babies delivered at Mbagala and Sinza hospitals. Screening was conducted at birth for all newborns whose mothers consented for their babies to participate in the study. However, we excluded all newborns with other illnesses that may lead to admission and/or blood transfusion such as infection (severe malaria, bacterial infection) and as well as development of fever at birth and prematurity with complications. We also excluded babies whose mothers did not consent to participate in the study.

## Establishment of birth cohort

### Municipal level

In order to ensure ownership, proper supervision, and management, any project or initiative that involves a health facility under a particular municipality in Tanzania, approval from the particular municipal office must be acquired before the initiation of the project. In this case, we received approval from the Temeke and Ubungo municipalities. We also received approvals for project staff to be allowed to take part in the study at the health facilities especially within the labor ward.

### Facility level: Project meetings

The medical officer in charge (MOI) at the health facility serves as the project's core host, giving all necessary instructions to ensure the project's successful operation. We first engaged with the MOIs at Mbagala and Sinza to introduce the project and together discussed the best practices that will allow the research to operate successfully, such as how best to engage the management of the facility, the leads of the respective units, required hospital staff, and the study participants. We then conducted a number of meetings with the hospital management and respective units which included Medical Officer In charge (MOI), heads of units, doctors, and nurses practicing at the Labor ward and Reproductive and Child Health (RCH) clinics. During the meeting, we introduced the project, highlighted the study protocol, and indicated the involved hospital sections including the labor, postnatal wards as well as RCH unit. We used these meetings to get inputs from the doctors and nurses on the best patient engagement approaches to be used during the enrollment and screening process as it deemed fit to the respective facility. Some of the issues that were highlighted in these meetings included the best staff-patient engagement approaches and remunerations. The investigators discussed matters relating to patient's beneficence, autonomy and the value of voluntariness in conducting the study. For the ward nurses that were directly involved in counseling and study activities, plans for remuneration were also discussed.

### Labor ward training

At the labor ward, nurses' training sessions were conducted on various aspects including (i) counseling before and after enrollment (ii) consenting process, and (iii) filling of the enrollment case report form (CRF). Practical sessions were also conducted on the collection of umbilical cord blood for SCD screening and storage for other laboratory assays.

### RCH training

Nurses' training at RCH was mainly focused on SCD counseling and health education as well as SCD screening using point of care test for babies of 6 weeks of age. We also provided training on dissemination of SCD screening results and counseling sessions for the mothers. Practical training sessions on sample collection and storage at the follow-up visits were conducted at each facility.

## Enrollment into the birth cohort

### Counseling of mothers at the labor ward

All mothers admitted at the labor ward, either in labor or not, received counseling to take part in the study. A trained project nurse provided education on SCD, neonatal SCD screening, and its significance in SCD management. The study nurse also utilized this opportunity to explain the importance of the study. The mothers were also informed that no cost of screening will be directed to them. All communications were conducted in Swahili language.

### Consenting of study participants

Following the counseling session, the mothers who agreed for their babies to take part in the study were required to consent before the screening process. A written informed consent in Swahili language was provided and mothers received information about the study including an explanation of how to participate in the study, possible risks, and benefits of participating in the study, and the freedom of choosing to or not to take part in the study. If they agreed to participate in the study, the participants were provided with the consent forms to sign. Exceptionally, for some pregnant women who were in active labor and were unable to sign the forms, oral informed consent was administered, followed by written consent post-delivery.

### Newborn screening for SCD

Immediately after delivery, the umbilical cord was clamped and umbilical cord vein was identified. In aseptic conditions, 5 ml of umbilical cord blood was collected and stored in ethylenediaminetetraacetic acid (EDTA) tubes. Out of the 5 ml, 2 ml was used for SCD screening using a point of care test, Hemotype SC, by following the manufacturer's procedure (Silver Lake Research Corporation, United State of America). The rest of the blood sample was processed and archived at the Hematology Clinical and Research Laboratory **(HCLR)** MUHAS for molecular and genetic assays at a later stage. For the babies who test positive for SCD, High performance Liquid Chromatography (HPLC) was used as a confirmatory SCD test.

### Enrollment of study participants into the study

Following delivery and stabilization of mother and baby, an enrollment form was filled. Trained research assistants collect clinical and socio-demographic information about the baby including; any presenting symptoms at birth and during newborn's examination, the health facility details (Hospital ID, etc), gestation age, baby's gender, and date of birth. In addition, parents' information such as parents' names, tribes, residence, and contact information was collected. Finally, sample collection details were also recorded. In order to ensure good compliance with the study, we focused our enrollment on babies whose families reside around the health facility (i.e., Mbagala and Sinza hospitals) as their babies were most likely attending immunization clinics at the same facilities. Following the completion of the enrollment process, the mothers were informed about the study follow-up plan for up to 3 years of age with the follow-up visits synchronized with the immunization schedule. The mothers were also informed of two additional visits of the study which were to be conducted at the age of 24 and 36 months.

## Maintenance of birth cohort

### Utilizing the immunization platform for follow up schedule

To maximize adherence, scheduling of the first six follow-up visits (1.5, 2.5, 3.5, 6, 9, 18 months) was done according to the immunization schedule (Table 1), working closely with the designated nurses at immunization units. The scheduling was synchronized and done in accordance to the Tanzania Expanded Program of Immunization (EPI) (Table 1). Collaboration with the hospital RCH unit encouraged compliance of the mothers to the study due to the respect, trust, and familiarity they had to the immunization program and the RCH services.

### Provision of clinical services through the immunization platform

During each follow up visit, we worked with the RCH nurses to ensure the following: (i) supervision of the enrolled babies to complete the vaccination process, (ii) synchronization of the subsequent immunization visit with that of the study follow up, (iii) blood sample collection for laboratory tests, and (iv) provision of clinical services for the babies including SCD and full blood count results feedback, clinical history documentation and counseling. Each baby who tested negative for SCD received their first results on the first visit day at the age of 42 days (6 weeks). For newborns who tested positive for SCD, home visits were done as soon as they were diagnosed to inform the parents of the test results. These visits were conducted by a doctor, and a nurse and followed up later by a research assistant. During these visits, parents were given SCD health education on; etiology of disease, presenting symptoms, possible danger signs, treatment and home care, and possible complications. In addition, the parents were also educated on the significance of early enrollment into care as part of disease management. Finally, scheduling of the first follow up visit at 6 weeks coupled with the first immunization was conducted.

### Follow up visit reminders

Before leaving the RCH clinic the mothers were reminded of the babies' next follow up visit. In addition, regular reminders through phone calls and text messages were often made to all study participants to enhance adherence to follow-up visits. A week before the scheduled clinic visit, the parents were reminded three times through calls and text messages to ensure their babies attend their follow-up visit and immunization clinic.

### Follow up visits processes

At the first follow up visit (6 weeks or 1.5 months), all mothers were guided for their newborns to receive their vaccination at the RCH clinics as per EPI. After vaccinations, a trained study nurse in collaboration with the RCH nurse were responsible for providing the mothers with their babies' results for SCD testing and CBC. In addition, the nurse utilized this visit to provide a refresher health education on SCD and the importance of the study. Clinical and demographic information was collected using standardized case report forms (CRF) for follow-up visits. As part of clinical monitoring of the babies, 2 milliliters of blood sample were collected for conducting laboratory tests. The laboratory assay included complete blood count (CBC), hemoglobin subtype quantification by HPLC, F cell count by flow cytometry, and archiving of samples for genomic studies considered to be conducted in the future on advanced therapy for patients with SCD. For the subsequent follow-up visits, the same procedures were conducted except for the SCD results feedback which was conducted once at the first visit. For all visits, severely deranged laboratory results were immediately communicated to the mothers and appropriate measures were taken to ensure that the baby received the required care. These cases were treated as emergencies and therefore results communication was performed prior to the scheduled follow up visits.

## Data management

Demographic and clinical information were collected using standardized CRFs and were subsequently entered into a Research Electronic data capture (REDCap): backed up with the MySQL relational database management system. Each participant was assigned a unique study number that was linked to their study information. Data access was restricted to the approved project staff who have received training in ethical data management with considerations to data privacy and security.

### Ethical consideration

This study received ethical approval from MUHAS Institutional Review Board (IRB) committee with reference number Ref.No.DA.282/298/01.C/. All study participants received a unique identifier that is used to link to other study information. All project data/files are secured and can only be accessed by designated study personnel.

## Results

### Establishment of the cohort

At the initiation of the study, we first engaged the top management of the hospitals. In the course of the study, we observed poor buy-in especially in the sections that were relevant to the study. Through consultations with the top management, we received guidance on how best to address this challenge. We then conducted meetings with the leaders of various units both directly or indirectly related to the study, resulting in the resolution of the challenges.

Our initial approach at the labor ward and RCH clinics was to involve only a few staff. However, this became challenging due to routine duty rosters in which different nurses would work at different work shifts. Similarly, through guidance by the management, we conducted refresher training for all staff leading to increased capacity for involvement in the study by all staff. This approach increased the study buy-in at all levels of the facility.

### Enrollment to the birth cohort

**Figure 2:**
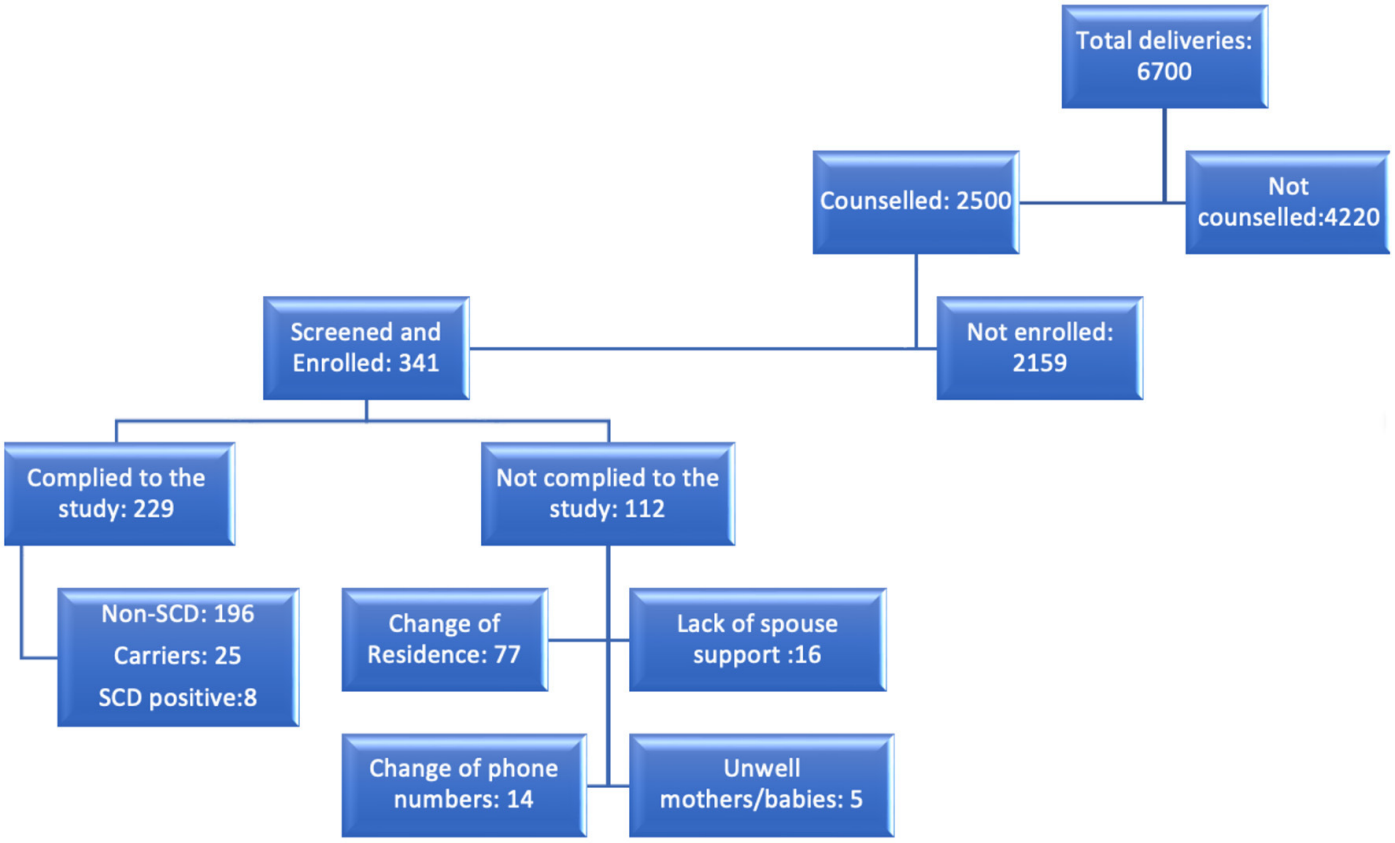
A flow chart showing the flow of events/steps and involved individuals on each of these. The deliveries reported here are between the period of January 2020 and September, 2021.

Our study projection was to enroll a total of 400 babies with and without SCD in equal proportions by 2021. To date, we have enrolled a total of 341 babies. The average weekly delivery capacity for Sinza and Mbagala is estimated to be 60–80, with Mbagala being on the higher side. Between the period of January 2020 and September 2021, there were a total of 6,700 deliveries (Figure 2). Of these, 37.3% of the pregnant women received SCD health education. This number was much higher than our sample size (400) and weekly targets. Nevertheless, we aimed at utilizing this opportunity to reach out to all the pregnant women with SCD health education regardless of the final deliberation of being enrolled into the study. Therefore, the lack of counseling for the 4,220 pregnant women was mainly because of shortage of staff and time as counseling was only done during the day while pregnant women were admitted and deliveries happened both during the day and night.

This delivery capacity was more than our weekly target which was set at 50 babies. In addition, despite the large delivery capacity of these facilities, our enrollment target was not met as planned. In this report, we describe some of the reasons/challenges that contributed to this.

First, provision of SCD health education followed by consenting, forms a critical part of this study. Initially, we attempted to conduct these two aspects of the study at the labor ward for already admitted pregnant women. This was challenging due to the fact that some of the pregnant women who were experiencing mild or active labor could not really follow through the process. This led to difficulties in obtaining consent which was important before delivery. We encountered challenges such as some of the pregnant women agreeing to consent while in active labor and later lacking memory to have done so. To address this challenge, all pregnant women who were in active labor were only informed about the study at the labor ward and if agreed, the proper counseling and signing of consent forms were done after delivery. Efforts were also made to ensure the counseling process is done earlier, during admission to the labor ward. This ensured the proper consenting process before the pregnant women progressed into active labor. We therefore agreed to focus on a few pregnant women and ensure a proper counseling process is conducted.

Second, complete filling of data in the enrollment case report forms (CRF) was necessary for this study to be successful. At enrollment, we expected to capture all basic and baseline information. We encountered a challenge of missing/wrongly entered details including contact and residence information. Due to this, we could not reach some of the mothers for follow-up reminders and hence we had to exclude their babies from the study. The main reason for this challenge was the high workload for the labor ward nurses. To address this challenge, a full-time study nurse was recruited to work closely with the labor ward nurses. We also used the staff credentials records (included in the CRF) to identify the nurses who may need further training or reinforcement on counseling and enrollment procedures.

Third, during the training we insisted on ensuring that enrolled babies are from families residing in the health facilities' catchment areas. We later established that a number of pregnant women (26) who consented for their babies to take part in the study were from upcountry and had visited the health facility only for delivery purposes. Some of the babies were enrolled in the program despite their families residing outside Dar es Salaam, some pregnant women were referred from other hospitals only for delivery and post-delivery care, while others resided around the mentioned facilities for socio-cultural reasons. This posed challenges during follow-up as many of them did not adhere to the follow-up schedule and hence their babies were excluded from the study.

Therefore, due to the sensitivity of this process, we had to review our targets and focus on the quality of the data rather than quantities leading to changes to our enrollment targets and timelines.

### Maintenance of the birth cohort

The ultimate goal of maintaining any birth cohort is to be able to follow up the babies successfully in order to monitor or evaluate a particular course. The purpose of this birth cohort was to follow up babies with and without SCD for a period of three years of age. To date, a total of 311, 186, 133, 81, 44 and 16 babies have returned to their 1, 2, 3, 4, 5, and 6 visits, respectively with 50–80% attrition rate (Table 2). We have also succeeded in maintaining 229 babies (Figure 2) who have returned to more than 3 follow up visits. Out of these, 196 are normal, 25 are carriers, and 8 babies with SCD.

**Table 2:** The number of babies who have attended follow up visits at Mbagala and Sinza.

	**Visit 1** **(1.5 months)**	**Visit 2** **(2.5 months)**	**Visit 3** **(3.5 months)**	**Visit 4 (6 months)**	**Visit 5 (9 months)**	**Visit 6 (18 months)**
Mbagala	253	163	120	72	39	14
Sinza	58	23	13	9	5	2
Total	**311**	**186**	**133**	**81**	**44**	**16**

The success of the study has been greatly influenced by utilizing the immunization platform. However, some of the babies missed their visits for various reasons.

The main challenge reported was a lack of understanding for some mothers. Despite adequate counseling before and after giving birth, some women were still hesitant to continue with follow-up, especially those with healthy babies which led to dropouts. To address this challenge, we continued with regular counseling and health education sessions which were provided during the follow up clinics at the RCH.

Ideally, the pre and post-delivery counseling sessions would have been more effective if provided to the expectant couples to ensure mutual understanding and consenting. As per the hospital and labor wards settings, the presence of spouses in the labor ward was not allowed, for that matter, counseling was done only to expectant mothers. This lack of spouse engagement and involvement during counseling had posed a challenge in maintaining some of the enrolled babies due to a lack of understanding of their partners.

Our study was designed to maximize the utilization of the RCH clinic. This approach has proven to work, however, since most RCH nurses trained for the follow-up activities are also involved in the vaccination activities at the RCH, they sometimes face challenges with the clients' workload. For this reason, the mothers with babies enrolled into the study had to wait longer to complete both vaccination and the follow up visit processes. To address this, we strengthened the team by hiring an additional study nurse to assist the RCH nurses. We also established a mechanism for the research assistant to assist the nurses in scheduling and documentation of visits.

Due to some of the reasons explained above, we experienced several dropouts; up to October 2021, we had a total of 112 dropouts from the cohort (Figure 2). Below are a number of attributable factors for the dropouts and loss to follow up.

Reasons for dropouts include (i) change of residence (77) reflecting gaps in the counseling process; some mothers and newborns reallocated after delivery and could not be traced at the respective RCH clinics (ii) spouses not supportive; Some spouses advised the mothers to drop out from the study due to lack of understanding, also some mothers feared that their partners would not agree to their involvement in the study (16) and (iii) change of phone numbers (14) (iv) mothers/babies being unwell (5). Some of these factors are continuously being addressed by enhancing health education to mothers and families where possible, establishing new ways of disseminating information such as digitalization of reminders and health education as well as general health education to the community.

## Discussion

This is among few reports on birth cohorts (14) established for SCD research in Africa. Here we have described the steps we undertook to establish a birth cohort which forms a strong basis for executing a larger study on genetic determinants of fetal hemoglobin decline and how this influences SCD expression in the first 3 years of life. SCD is a public health condition that has been recognized by WHO (15). Unfortunately, most of the affected individuals are babies under 5 years of age residing in sub-Saharan Africa. SCD contributes significantly to under five mortality rates (3) in many affected African countries (16), SCD is now included in the strategic plans to prevent and control Non-Communicable Diseases (NCDs) in most African countries, Tanzania included (17).

Newborn screening (NBS) coupled with comprehensive care is proven to reduce mortality and morbidity caused by SCD (18–20). Unfortunately, most African countries have not been able to establish national NBS programs due to various reasons including cost and limited human and logistical resources (21). However, there is currently a great push to establish sustainable national NBS programs that will screen for a number of conditions including SCD. This is a great initiative which will allow early interventions and reduction of both morbidity and mortality contributed by SCD.

Successful NBS are those coupled with comprehensive care as well as early childhood research to better understand the mechanisms of disease expression, management, and treatment outcomes. One of the approaches to achieve this is to establish manageable birth cohorts that can be followed up for a period of time, preferable up to five years (22).

In this report, we have documented the process we undertook for establishing a birth cohort in Tanzania (2019-to date). In order to better understand SCD expression in babies, we set to follow a cohort of babies with and without SCD for a period of 3 years. Based on the estimated SCD prevalence (0.8%) in Dar es Salaam, Tanzania we expected to recruit babies without SCD much faster than those with SCD. We therefore coupled our study with two existing platforms NBS and the immunization program. We also anticipated low compliance to follow up visits especially for babies without SCD due to the fact these babies are not sick and the parents may not see the necessity of participating in such a study. It was therefore necessary to establish such a cohort on a robust and sustainable early childhood program, in this case the national immunization platform which has proven to be successful. Therefore, we attempted to synchronize as much as possible all the aspects of the study to the immunization program including identifying study sites with immunization services, synchronizing the follow up study visits to follow the immunization schedule and utilizing the RCH resources (personnel and infrastructure).

Through this work, we have learnt to tap into expertise and experience that health facilities have in taking part in research projects. We observed that conducting the initiation and feedback/progress meetings increased the project buy-in at the health facilities and enhanced a smooth addressing of the challenges. It is clear that the health facilities are overburdened by the routine healthcare provision, however, in most cases research is also accommodated especially when there is a direct involvement of the staff.

It was notable that the staff based at the facility understood much better the language and the communication/social-cultural barriers with the study participants (local mothers/families) and hence their involvement in the study helped to address some of these challenges. Although it was important to have a full-time study nurse at the health facility, it was necessary for the nurse to work closely with the in-house nurses. This was experienced both at the labor ward and the RCH clinics. Therefore, since our study success largely depended on site personnel at the labor ward and RCH, we started building the capacity of these nurses especially on SCD health education, counseling for SCD screening and enrollment into the study. Most of the nurses were receptive to the SCD knowledge, and it was more so for the nurses who had encountered SCD patients either at the hospital or at personal level. We chose a training mode which allowed interaction and hands on experience for both counseling and sample collection.

Our initial enrollment strategy was to target the pregnant women who were already admitted to the labor ward. The main reason for this was to increase the chances of dealing only with the pregnant women who will end up delivering at that site. Both Mbagala and Sinza sites are regional hospitals and hence in case of any indicators of complicated deliveries the pregnant women were referred to the next tier of referral hospitals. However, it was later evident that the labor ward was not a conducive environment for conducting health education since some pregnant women may be in active labor. Our second approach which was implemented at the admission stage worked better in terms of having a calmer environment for provision of both SCD health education and counseling of the pregnant women. During the course of providing SCD health education we observed that dealing with pregnant women who had prior SCD knowledge was much easier than those without. For this reason, we endeavored to provide health education to all pregnant women regardless of the ultimate enrollment status of their babies in the study. Compared to our study sample size of 400, health education was provided for a much higher proportion of pregnant women (Figure 2).

One of the challenges faced during the establishment of this birth cohort is couples' collective involvement during the consenting process. In our context, the presence of male spouses in the labor ward is not conveniently allowed, especially in public hospitals. This posed a challenge to partners' involvement in the consenting process which later on led to dropouts following the couple's disagreement and lack of proper counseling for men. This cultural aspect is also reflected in the antenatal clinic attendance in the country, where it is most common for women to attend antenatal clinics (ANC) without their partners (23). Traditional gender roles for males hinder their direct involvement in pregnancy and childbirth (24) and hence limit their general understanding of matters pertaining to babies let alone research issues. We therefore attempted to conduct home visits and phone calling to provide education to the partners of the mothers involved in the study. This has proven to work; however, it is anticipated to bear many fruits if it will be done at the enrollment stage.

For follow up purposes, contact details were collected from expecting mothers for close monitoring, immediate provision of results if needed and for reminding the parents to bring back the baby to attend both immunization and study visits which were synchronized. Considering the context in the labor ward, most mothers were not in possession of their phones, making the process prone to errors and the possibility of missing contact details from the mothers which led to some defaults. To address this challenge, we consulted with hospital records in order to fill in some of the missing information. This shows the importance of linking study and hospital databases. It also shows the importance of strengthening our hospital databases to capture demographic information that may be relevant for research purposes.

Since the enrollment involved a number of activities, insufficient time for proper counseling was also one of the challenges that hindered the proper recording of contact details and filling of the CRFs. This challenge was mostly addressed by the cooperation between the study and the hospital nurses. Although it was necessary to organize and clarify roles between the study and hospital nurses, the cooperative atmosphere built the capacity for research for both sides.

Geographical reallocation after delivery was another factor that led to a loss to follow up, this challenge for maintaining birth cohorts has been reported elsewhere (25). Although this is a common practice in our cultural context, it should be handled well-during counseling and enrollment such that sufficient information is gathered to avoid study dropouts. Again, the cooperation between the study and the hospital nurses during counseling and enrollment assisted in addressing this challenge for our study.

Our study follow-up visits were impacted positively by the utilization of the immunization platform. Establishing birth cohorts and conducting newborn studies using local and traditional setting and contexts is not a popular method except in Nigeria and Ghana (9). However, since most of our study participants were babies without SCD, linking their study visits to their immunization clinics increased compliance to the study. We also ensured that those babies enrolled into the study received immunization services in a fast-tracked manner before being attended by the project team so as to reduce the turnaround time. In cases where the project doctor observed a situation that required additional clinical attention, the hospital pediatricians were informed so as to provide the necessary care to the baby. This way the integration of the study and hospital services were more benefitting to the study participants and hence increased compliance to the study.

Our integration with the NBS program has also been productive both ways. On one hand our study has enrolled SCD positive babies who have been screened through NBS. On the other hand, babies who have been screened through the study have been also enrolled into the NBS program for further follow up and planning of the comprehensive care.

Our study is exemplary of the importance of integrating early childhood programs for building research that will inform early childhood events, especially in limited resource settings. In this case we have utilized the integration of our study with existing NBS and immunization programme. Many African and other low-income countries are still facing challenges of sustaining NBS services. This is largely because of limited logistical and human resources because they tend to be costed and managed independently. One approach that can address these challenges is utilization of immunization programs as reported previously in Nigeria by Nnodu (6). In addition, further integration of early childhood research programs on such platforms as the immunization program increase not just compliance to the study but also sustainability and utilization of research findings.

Despite the crucial role of birth cohort especially in genomic studies, establishing birth cohort is prone to high rates of attritions (14) which has also been the case in this study. This approach however, is feasible and implementable in resource limited settings.

## Conclusion

To the best of our knowledge, this is among the first reports that describe the utilization of immunization platforms for establishment of the birth cohort with a component of longitudinal follow up of more than two years. This is an excellent platform for establishment of birth cohorts especially in places with limited resources. It is important to enhance the research capacity of the RCH units including the immunization platforms especially for early childhood programs. In the coming years, research training for RCH nurses will be useful especially for incorporating pilot childhood research. For SCD, this is especially important for both screening and follow up of babies with SCD. It is therefore important to continue the exploration of better ways of building the research capacity of this important early childhood section. Our study has provided a proof of concept of how immunization platforms can be utilized for research especially those related to birth cohorts.

## Data availability statement

The original contributions presented in the study are included in the article/supplementary material, further inquiries can be directed to the corresponding author.

## Ethics statement

The studies involving human participants were reviewed and approved by Muhimbili University of Health and Allied Sciences Institutional Review Board. Written informed consent to participate in this study was provided by the participants' legal guardian/next of kin.

## Author contributions

SiN and DN were involved in conceptualization, proposal development, study participant enrollment, data collection, data analysis, interpretation, and manuscript drafting. BN, FK, GN, DS, and SaN participated in conceptualization, data interpretation, and manuscript preparation and revision. EM, MM, and JM participated in conceptualization and manuscript revision. All authors read and approved the final version of this manuscript.

## Funding

Research reported in this publication was supported by the Fogarty International Center of the National Institutes of Health under Award Number K43TW011167.

## Conflict of interest

The authors declare that the research was conducted in the absence of any commercial or financial relationships that could be construed as a potential conflict of interest.

## Publisher's note

All claims expressed in this article are solely those of the authors and do not necessarily represent those of their affiliated organizations, or those of the publisher, the editors and the reviewers. Any product that may be evaluated in this article, or claim that may be made by its manufacturer, is not guaranteed or endorsed by the publisher.
